# Supplementary figures and images for: The vgll3 Locus Controls Age at Maturity in Wild and Domesticated Atlantic Salmon (Salmo salar L.) Males
Source: PLoS Genet. 2015 Nov 9;11(11):e1005628. doi: 10.1371/journal.pgen.1005628 (PMC4638356; doi:10.1371/journal.pgen.1005628)

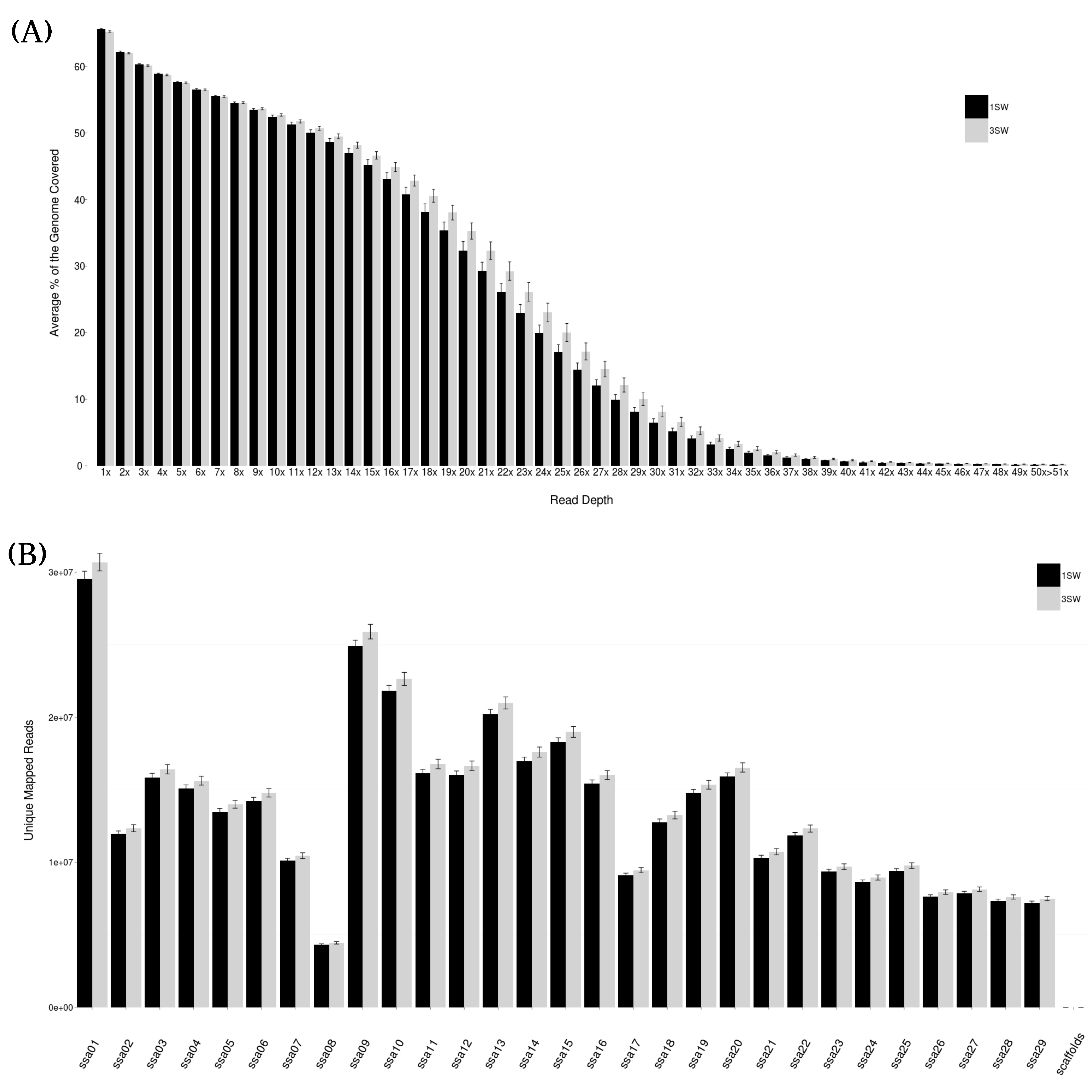

Supplement: S1 Fig — (A) Average read depth in all pooled samples (x-axis). The y-axis is showing average percent of the genome covered, with error bars. (B) Average number of uniquely mapped sequences (y-axis) on each chromosome (x-axis), with error bars. “Scaffolds” refers to unplaced contigs in the current genome assembly. (TIF) [file pgen.1005628.s001.tif]

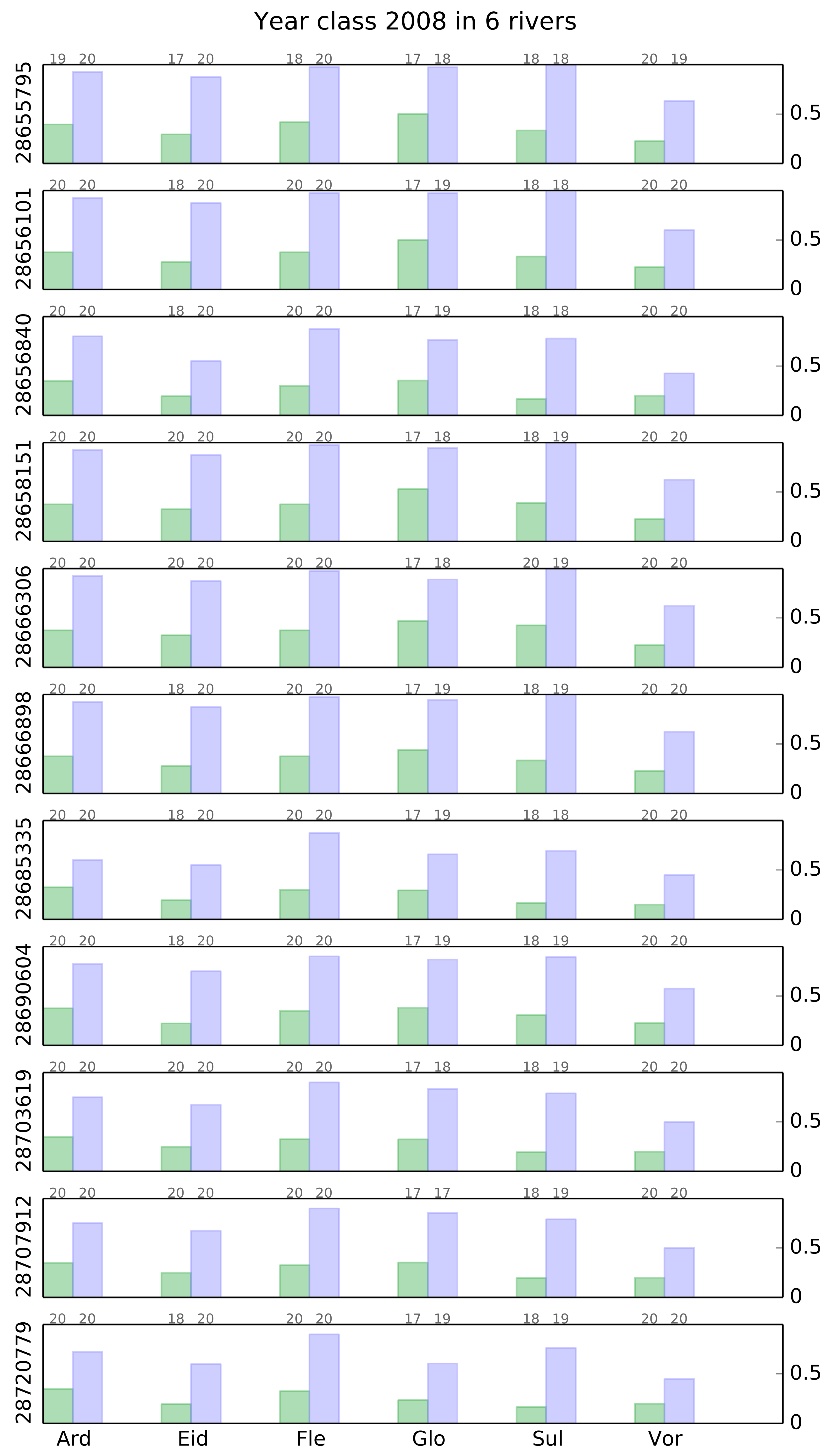

Supplement: S2 Fig — Frequencies of the late maturation allele in year class 2008 are shown for 1SW (green bars) and 3SW (blue bars) fish. The position of each SNP in Chr 25 is shown in the leftmost part. Above each bar the number genotyped fish is indicated. The y-axis shows the allele frequency between 0 and 1. Abbreviations; Ard—Årdalselven, Eid—Eidselven, Fle—Flekkeelven, Glo—Gloppenelven, Sul—Suldalslågen and Vor—Vormo. (TIF) [file pgen.1005628.s002.tif]

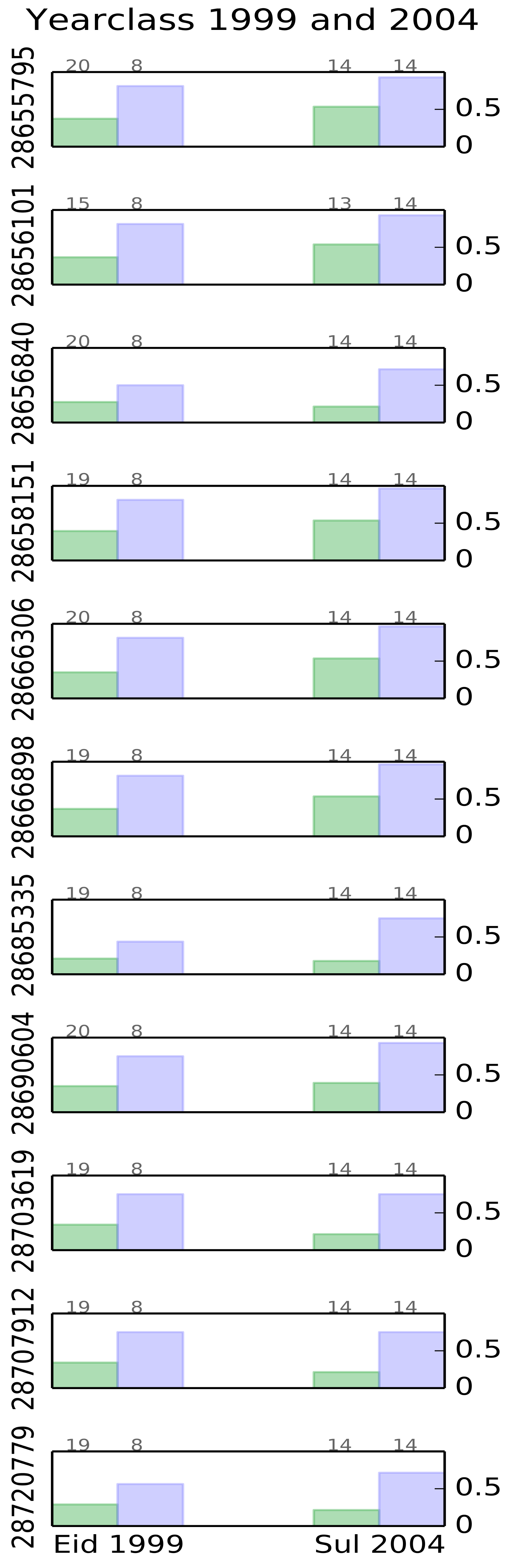

Supplement: S3 Fig — Frequencies of the late maturation allele in Eidselva year class 1999 and Suldalslågen yearclass 2004 are shown for 1SW (green bars) and 3SW (blue bars) fish. The position of each SNP in Chr 25 is shown in the leftmost part. Above each bar the number genotyped fish is indicated. The y-axis shows the allele frequency between 0 and 1. Abbreviations; Eid—Eidselven, and Sul—Suldalslågen. (TIF) [file pgen.1005628.s003.tif]

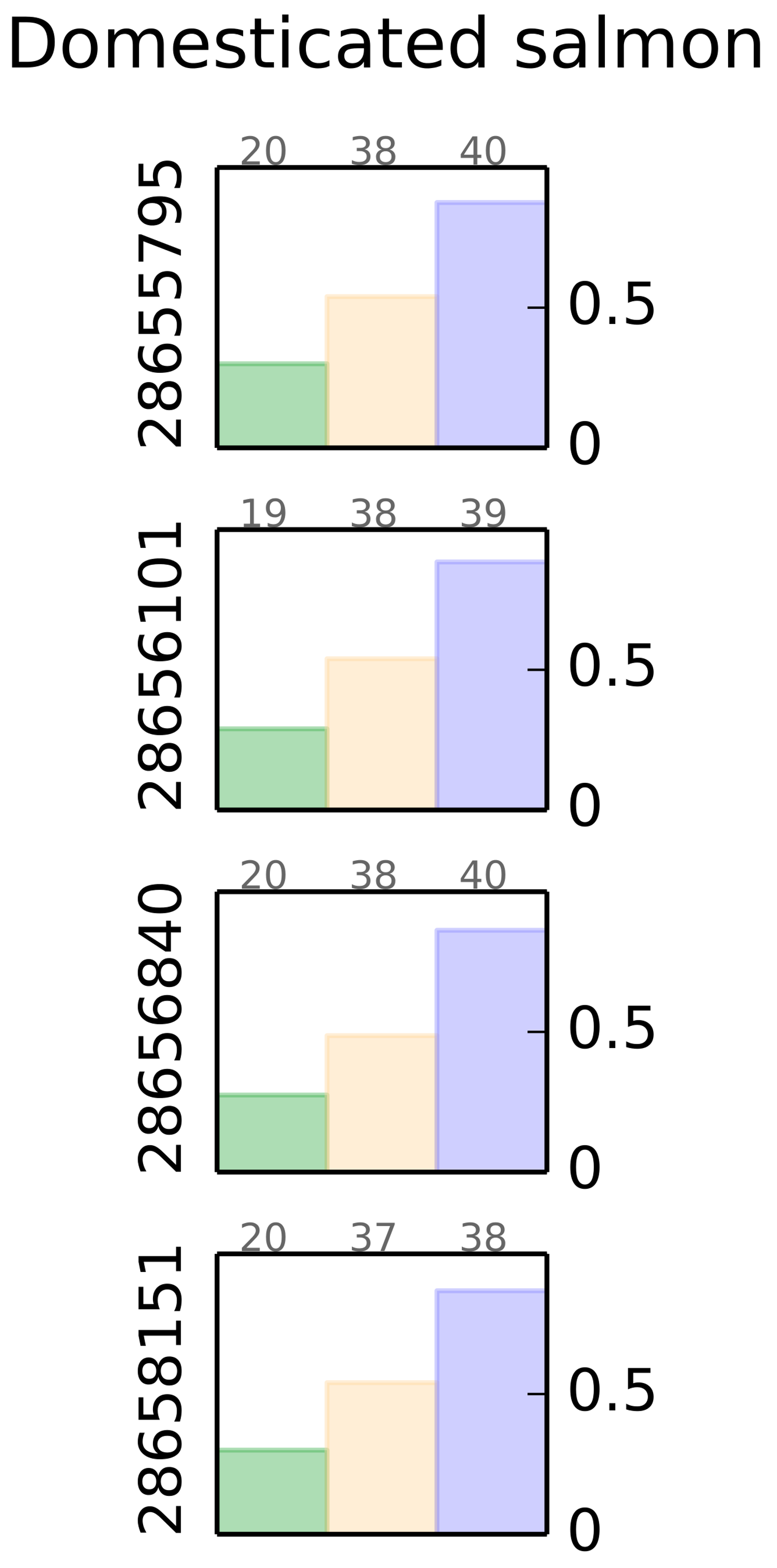

Supplement: S4 Fig — Frequencies of the late maturation allele in farmed salmon (Mowi strain) strains, maturing either after 1SW (green bars), 2SW (yellow bars) or after 3SW or more in sea water (blue bars). The position of each SNP in Chr 25 is shown in the leftmost part. Above each bar the number genotyped fish is indicated. The y-axis shows the allele frequency between 0 and 1. (TIF) [file pgen.1005628.s004.tif]
