# Supplementary material for: The vgll3 Locus Controls Age at Maturity in Wild and Domesticated Atlantic Salmon (Salmo salar L.) Males
Source: PLoS Genet. 2015 Nov 9;11(11):e1005628. doi: 10.1371/journal.pgen.1005628 (PMC4638356; doi:10.1371/journal.pgen.1005628)
Supplement: S1 Table — Table of SNPs significantly associated to age at maturity. The table includes scaffold names, chromosome position, reference/alternative (Ref/Alt) Alleles, Average Coverage (+/-SE) and P-values. (PDF) [file pgen.1005628.s005.pdf]

Scaffold	Chromosome	Position	Ref/Alt Allele	Average Coverage (+/-SE)	P_value
gi|829620127|gb|CM003279.1|	ssa01	31287379	G/A	27.25 (+/-1.70)	4.39E-09
gi|829620127|gb|CM003279.1|	ssa01	46456741	T/C	21.92 (+/-3.59)	7.39E-10
gi|829620127|gb|CM003279.1|	ssa01	49673108	A/G	20.92 (+/-1.44)	1.22E-08
gi|829620127|gb|CM003279.1|	ssa01	103676254	T/C	23.08 (+/-2.83)	3.75E-10
gi|829620127|gb|CM003279.1|	ssa01	145725963	T/G	24.00 (+/-1.45)	1.15E-09
gi|829620127|gb|CM003279.1|	ssa01	148212386	C/G	26.67 (+/-1.03)	3.15E-09
gi|830106998|gb|CM003280.1|	ssa02	14793139	C/T	24.33 (+/-2.05)	6.62E-10
gi|830107118|gb|CM003281.1|	ssa03	51197942	T/A	21.67 (+/-1.56)	3.09E-08
gi|830107176|gb|CM003282.1|	ssa04	19451064	C/T	22.58 (+/-1.67)	1.10E-08
gi|830107225|gb|CM003283.1|	ssa05	58756652	G/A	20.50 (+/-2.11)	2.94E-08
gi|830107225|gb|CM003283.1|	ssa05	66775778	C/T	25.58 (+/-1.57)	2.89E-08
gi|830107324|gb|CM003284.1|	ssa06	11068702	T/A	21.83 (+/-1.00)	3.10E-08
gi|830107324|gb|CM003284.1|	ssa06	41741034	T/G	27.08 (+/-1.47)	4.84E-10
gi|830107324|gb|CM003284.1|	ssa06	41741035	T/A	27.08 (+/-1.51)	3.07E-10
gi|830107324|gb|CM003284.1|	ssa06	57854788	C/A	19.92 (+/-1.37)	1.06E-08
gi|830107404|gb|CM003285.1|	ssa07	11591650	A/G	23.83 (+/-1.70)	1.32E-09
gi|830107404|gb|CM003285.1|	ssa07	52882119	C/T	30.42 (+/-2.47)	1.19E-08
gi|830107505|gb|CM003287.1|	ssa09	51209103	G/A	26.25 (+/-1.49)	9.30E-09
gi|830107530|gb|CM003288.1|	ssa10	70868455	G/T	23.42 (+/-1.88)	5.52E-09
gi|830107530|gb|CM003288.1|	ssa10	89190499	A/G	17.17 (+/-1.30)	1.74E-08
gi|830107530|gb|CM003288.1|	ssa10	89190500	T/C	17.08 (+/-1.25)	5.25E-09
gi|830107530|gb|CM003288.1|	ssa10	89190503	T/A	17.00 (+/-1.35)	7.92E-09
gi|830107628|gb|CM003289.1|	ssa11	2923483	G/A	15.08 (+/-0.79)	1.61E-09
gi|830107628|gb|CM003289.1|	ssa11	4217691	A/C	22.50 (+/-1.89)	2.61E-08
gi|830107628|gb|CM003289.1|	ssa11	5963544	C/T	13.42 (+/-1.09)	2.16E-08
gi|830107711|gb|CM003290.1|	ssa12	18949353	G/T	20.17 (+/-1.65)	3.26E-13
gi|830107711|gb|CM003290.1|	ssa12	18949371	C/T	16.50 (+/-1.26)	1.15E-09
gi|830107711|gb|CM003290.1|	ssa12	21631791	A/C	21.83 (+/-2.00)	2.48E-08
gi|830107711|gb|CM003290.1|	ssa12	43211270	G/T	22.67 (+/-1.77)	2.40E-08
gi|830107711|gb|CM003290.1|	ssa12	64945518	T/C	22.92 (+/-1.31)	1.88E-09
gi|830107850|gb|CM003291.1|	ssa13	23247182	A/T	24.58 (+/-1.83)	4.38E-09
gi|830107850|gb|CM003291.1|	ssa13	37319811	C/A	20.25 (+/-1.21)	1.49E-08
gi|830107850|gb|CM003291.1|	ssa13	45158241	C/T	20.67 (+/-1.59)	7.14E-10
gi|830108056|gb|CM003292.1|	ssa14	22883451	G/A	25.08 (+/-2.17)	2.94E-08
gi|830108056|gb|CM003292.1|	ssa14	32404390	T/A	21.75 (+/-1.41)	2.36E-08
gi|830108165|gb|CM003293.1|	ssa15	38122288	C/T	26.25 (+/-1.70)	1.06E-08
gi|830108165|gb|CM003293.1|	ssa15	38146809	T/C	22.58 (+/-1.76)	3.22E-09
gi|830108333|gb|CM003294.1|	ssa16	34583743	G/A	14.42 (+/-1.12)	1.59E-08
gi|830108333|gb|CM003294.1|	ssa16	35773334	C/T	23.17 (+/-2.02)	6.46E-09
gi|830108441|gb|CM003295.1|	ssa17	1382577	A/T	19.50 (+/-2.05)	3.01E-08
gi|830108441|gb|CM003295.1|	ssa17	40512817	G/A	23.50 (+/-1.44)	5.16E-12
gi|830108544|gb|CM003296.1|	ssa18	20282643	T/G	25.50 (+/-1.24)	1.41E-08
gi|830108544|gb|CM003296.1|	ssa18	44343380	T/G	23.08 (+/-1.87)	1.32E-08
gi|830108544|gb|CM003296.1|	ssa18	67442791	A/G	21.58 (+/-1.97)	3.68E-09
gi|830108665|gb|CM003297.1|	ssa19	22108510	T/C	23.83 (+/-1.34)	2.80E-08
gi|830108665|gb|CM003297.1|	ssa19	25171855	T/C	25.75 (+/-1.14)	1.75E-08
gi|830108900|gb|CM003298.1|	ssa20	14765877	T/C	22.75 (+/-1.28)	6.47E-09
gi|830108900|gb|CM003298.1|	ssa20	29231934	C/A	21.17 (+/-1.93)	5.42E-09
gi|830109011|gb|CM003299.1|	ssa21	8546730	T/C	22.92 (+/-2.36)	1.25E-12
gi|830109074|gb|CM003300.1|	ssa22	27267010	T/C	24.42 (+/-1.26)	7.35E-10
gi|830109129|gb|CM003301.1|	ssa23	11346449	T/G	21.00 (+/-0.97)	7.58E-09
gi|830109129|gb|CM003301.1|	ssa23	20613796	C/T	27.83 (+/-1.66)	4.14E-09
gi|830109129|gb|CM003301.1|	ssa23	20618934	C/A	22.58 (+/-1.10)	2.37E-08
gi|830109129|gb|CM003301.1|	ssa23	25405000	C/A	23.50 (+/-1.51)	1.46E-08
gi|830109254|gb|CM003302.1|	ssa24	17853327	G/T	20.17 (+/-1.60)	2.34E-08
gi|830109323|gb|CM003303.1|	ssa25	19542862	T/C	26.17 (+/-1.90)	4.34E-09
gi|830109323|gb|CM003303.1|	ssa25	28542944	A/T	19.50 (+/-1.48)	5.47E-09
gi|830109323|gb|CM003303.1|	ssa25	28561928	G/C	22.75 (+/-1.81)	5.83E-16
gi|830109323|gb|CM003303.1|	ssa25	28561929	C/T	22.58 (+/-1.83)	1.05E-14
gi|830109323|gb|CM003303.1|	ssa25	28563202	G/C	23.42 (+/-1.36)	4.04E-12
gi|830109323|gb|CM003303.1|	ssa25	28655795	T/C	24.75 (+/-1.26)	2.12E-19
gi|830109323|gb|CM003303.1|	ssa25	28656101	T/C	19.50 (+/-1.53)	3.18E-16
gi|830109323|gb|CM003303.1|	ssa25	28656840	C/T	22.50 (+/-1.69)	3.19E-10
gi|830109323|gb|CM003303.1|	ssa25	28658151	C/G	13.33 (+/-0.99)	9.36E-13
gi|830109323|gb|CM003303.1|	ssa25	28661044	T/G	19.50 (+/-1.24)	8.93E-18
gi|830109323|gb|CM003303.1|	ssa25	28661957	G/A	19.92 (+/-1.54)	5.86E-20
gi|830109323|gb|CM003303.1|	ssa25	28662430	T/C	23.08 (+/-1.59)	5.39E-14
gi|830109323|gb|CM003303.1|	ssa25	28662502	A/G	23.83 (+/-2.00)	7.41E-22
gi|830109323|gb|CM003303.1|	ssa25	28662646	G/T	25.25 (+/-1.64)	8.50E-14
gi|830109323|gb|CM003303.1|	ssa25	28664417	G/A	21.17 (+/-1.59)	2.53E-18
gi|830109323|gb|CM003303.1|	ssa25	28665717	A/C	18.08 (+/-1.02)	1.58E-09
gi|830109323|gb|CM003303.1|	ssa25	28666061	T/A	17.25 (+/-1.58)	5.04E-15
gi|830109323|gb|CM003303.1|	ssa25	28666141	C/A	17.00 (+/-1.31)	1.50E-11
gi|830109323|gb|CM003303.1|	ssa25	28666148	A/T	17.25 (+/-1.52)	1.22E-13
gi|830109323|gb|CM003303.1|	ssa25	28666155	C/T	17.33 (+/-1.79)	3.94E-12
gi|830109323|gb|CM003303.1|	ssa25	28666306	C/A	20.67 (+/-1.70)	6.64E-20

gi|830109323|gb|CM003303.1|	ssa25	28666898	A/C	24.42 (+/-1.08)	6.20E-26
gi|830109323|gb|CM003303.1|	ssa25	28667829	A/C	15.67 (+/-1.50)	8.72E-12
gi|830109323|gb|CM003303.1|	ssa25	28668344	A/C	22.25 (+/-1.09)	4.43E-18
gi|830109323|gb|CM003303.1|	ssa25	28668495	T/A	21.42 (+/-1.33)	2.25E-19
gi|830109323|gb|CM003303.1|	ssa25	28669006	G/C	20.42 (+/-1.14)	1.79E-17
gi|830109323|gb|CM003303.1|	ssa25	28669313	G/A	20.00 (+/-1.62)	4.69E-23
gi|830109323|gb|CM003303.1|	ssa25	28669319	C/T	20.08 (+/-1.73)	1.08E-18
gi|830109323|gb|CM003303.1|	ssa25	28669350	T/G	20.25 (+/-1.50)	1.42E-25
gi|830109323|gb|CM003303.1|	ssa25	28669434	T/C	18.00 (+/-1.79)	6.95E-21
gi|830109323|gb|CM003303.1|	ssa25	28669653	C/T	19.08 (+/-0.89)	1.44E-15
gi|830109323|gb|CM003303.1|	ssa25	28669853	A/G	23.50 (+/-1.90)	3.03E-18
gi|830109323|gb|CM003303.1|	ssa25	28674458	A/G	22.67 (+/-1.50)	2.70E-08
gi|830109323|gb|CM003303.1|	ssa25	28680099	C/T	24.92 (+/-2.04)	1.96E-08
gi|830109323|gb|CM003303.1|	ssa25	28684598	G/C	20.50 (+/-1.44)	2.61E-09
gi|830109323|gb|CM003303.1|	ssa25	28685335	C/T	26.83 (+/-2.16)	5.40E-15
gi|830109323|gb|CM003303.1|	ssa25	28687604	G/A	15.00 (+/-0.96)	9.76E-10
gi|830109323|gb|CM003303.1|	ssa25	28690604	A/C	25.08 (+/-1.77)	5.99E-19
gi|830109323|gb|CM003303.1|	ssa25	28691694	T/A	23.92 (+/-1.61)	1.86E-17
gi|830109323|gb|CM003303.1|	ssa25	28692147	T/G	14.08 (+/-0.75)	4.41E-09
gi|830109323|gb|CM003303.1|	ssa25	28692149	G/A	14.17 (+/-0.73)	5.72E-09
gi|830109323|gb|CM003303.1|	ssa25	28696354	A/G	23.33 (+/-1.86)	8.69E-17
gi|830109323|gb|CM003303.1|	ssa25	28696402	G/C	23.83 (+/-2.12)	2.17E-17
gi|830109323|gb|CM003303.1|	ssa25	28701719	A/G	23.33 (+/-1.72)	4.80E-18
gi|830109323|gb|CM003303.1|	ssa25	28702377	C/A	21.92 (+/-1.35)	9.07E-12
gi|830109323|gb|CM003303.1|	ssa25	28703367	G/C	15.33 (+/-1.25)	1.04E-13
gi|830109323|gb|CM003303.1|	ssa25	28703619	C/A	25.33 (+/-1.25)	6.99E-20
gi|830109323|gb|CM003303.1|	ssa25	28704581	A/G	24.17 (+/-1.88)	1.12E-12
gi|830109323|gb|CM003303.1|	ssa25	28707912	C/A	24.25 (+/-1.35)	4.39E-20
gi|830109323|gb|CM003303.1|	ssa25	28708791	T/C	23.50 (+/-2.17)	1.29E-14
gi|830109323|gb|CM003303.1|	ssa25	28709894	A/T	27.58 (+/-1.49)	6.83E-11
gi|830109323|gb|CM003303.1|	ssa25	28711976	G/T	26.42 (+/-1.42)	2.19E-08
gi|830109323|gb|CM003303.1|	ssa25	28713527	G/A	22.25 (+/-1.92)	1.18E-15
gi|830109323|gb|CM003303.1|	ssa25	28713925	A/G	22.83 (+/-1.35)	4.68E-14
gi|830109323|gb|CM003303.1|	ssa25	28715302	T/G	24.42 (+/-1.20)	1.14E-10
gi|830109323|gb|CM003303.1|	ssa25	28718057	A/T	23.33 (+/-1.09)	3.27E-16
gi|830109323|gb|CM003303.1|	ssa25	28720779	G/A	25.50 (+/-1.83)	3.25E-16
gi|830109323|gb|CM003303.1|	ssa25	28731586	C/T	23.42 (+/-1.44)	4.71E-10
gi|830109323|gb|CM003303.1|	ssa25	28738584	A/G	21.33 (+/-1.26)	3.75E-13
gi|830109323|gb|CM003303.1|	ssa25	28738988	C/T	19.08 (+/-1.09)	2.41E-08
gi|830109323|gb|CM003303.1|	ssa25	28752011	C/T	23.50 (+/-0.98)	2.15E-11
gi|830109323|gb|CM003303.1|	ssa25	28768099	T/G	22.75 (+/-1.67)	7.31E-09
gi|830109323|gb|CM003303.1|	ssa25	28909353	T/C	21.75 (+/-1.74)	2.70E-19
gi|830109323|gb|CM003303.1|	ssa25	28909449	G/C	21.50 (+/-1.73)	1.71E-13
gi|830109323|gb|CM003303.1|	ssa25	28909458	A/C	20.92 (+/-1.49)	1.46E-11
gi|830109323|gb|CM003303.1|	ssa25	28909468	T/G	21.17 (+/-1.42)	7.47E-14
gi|830109323|gb|CM003303.1|	ssa25	28909836	G/A	23.83 (+/-1.61)	2.93E-18
gi|830109323|gb|CM003303.1|	ssa25	28910202	T/C	19.08 (+/-1.54)	4.69E-16
gi|830109323|gb|CM003303.1|	ssa25	28913500	C/G	20.33 (+/-1.01)	1.09E-11
gi|830109323|gb|CM003303.1|	ssa25	28913551	T/A	23.92 (+/-1.49)	2.13E-15
gi|830109323|gb|CM003303.1|	ssa25	28914619	A/G	24.25 (+/-1.48)	4.78E-09
gi|830109323|gb|CM003303.1|	ssa25	28921536	G/C	22.08 (+/-1.47)	2.57E-09
gi|830109323|gb|CM003303.1|	ssa25	28921541	G/T	22.58 (+/-1.56)	2.33E-08
gi|830109323|gb|CM003303.1|	ssa25	31968523	G/T	19.92 (+/-1.55)	1.15E-08
gi|830109444|gb|CM003305.1|	ssa27	29689677	A/G	24.33 (+/-2.03)	5.04E-10
gi|830109458|gb|CM003306.1|	ssa28	15142009	C/T	23.33 (+/-1.97)	2.73E-10
gi|830109493|gb|CM003307.1|	ssa29	18296783	G/A	20.33 (+/-1.68)	1.91E-08
gb|AGKD04011258.1|	unplaced	38117	G/T	17.50 (+/-2.58)	2.13E-11
gb|AGKD04016318.1|	unplaced	10152	A/C	20.00 (+/-1.76)	2.53E-09
gb|AGKD04017001.1|	unplaced	9907	C/T	21.17 (+/-1.38)	1.01E-12
gb|AGKD04040582.1|	unplaced	784	A/T	13.33 (+/-1.79)	1.04E-10
gb|AGKD04130135.1|	unplaced	602	T/G	17.67 (+/-2.39)	2.94E-10
gb|AGKD04130135.1|	unplaced	616	C/G	15.08 (+/-1.60)	8.18E-09
