# Supplementary material for: The vgll3 Locus Controls Age at Maturity in Wild and Domesticated Atlantic Salmon (Salmo salar L.) Males
Source: PLoS Genet. 2015 Nov 9;11(11):e1005628. doi: 10.1371/journal.pgen.1005628 (PMC4638356; doi:10.1371/journal.pgen.1005628)
Supplement: S2 Table — (PDF) [file pgen.1005628.s006.pdf]

SNPs ID	Forward Primer	Reverse Primer	UEP_SEQ
ssa25_28655795	acgttggatgACTTTCACCAGAACACACGC	acgttggatgCGAATCATCTTTACAGACCG	cCCGAAGTTTATCCAAAAAGTTGTAG
ssa25_28656101	acgttggatgAGCTGGGTGTTTACAGTAGG	acgttggatgACGCTGCTGTTGCTGTCTC	ccctCCTGGAAACTGCTGCTCC
ssa25_28656840	acgttggatgTGCAGAACTGTTCATGACAC	acgttggatgGTAGAACGGGAAATAAGCTG	ACCTGTTAAAAGTTCTCTCC
ssa25_28658151	ACGTTGGATGAGCCCAGGGATACACAGTGA	ACGTTGGATGCATGGGTGTGTGTAGAGCAG	GGCTGGCCTGCTCCACCTCTGT
ssa25_28666306	ACGTTGGATGGCCCTTTATAGGTTTTCCAG	ACGTTGGATGCCTCTCCTCTCCTCCTGTC	TCTCCTCCTGTCCTCTCC
ssa25_28666898	acgttggatgGTTTCTCCTCTGTTGTCATC	acgttggatgAACCCAATCAGACCACACAG	ctcccCTGTTGTCATCCAGAATTAATC
ssa25_28685335	acgttggatgACAGGGAGAGGAGCCACCTT	acgttggatgTCAGGTTGTAACACAACAAG	AGCCACCTTCGGTAGAAGT
ssa25_28690604	acgttggatgTGAAGGTATCTGTGACCAAC	acgttggatgGCCTGTATCGTAACAAAATG	aTGCATTCGGAAAGAATTCAGA
ssa25_28703619	ACGTTGGATGGATACTACGCCCAGCTTTTC	ACGTTGGATGTTCTTTCCTCACTCCTGAGC	CTGAGCTCAGTTAACCG
ssa25_28707912	ACGTTGGATGTCAACTTAACCCACTCTCCC	ACGTTGGATGCATCACAGCTATGCCAGTTC	gggGAAGCAGTTGGATTTTCAA
ssa25_28720779	acgttggatgCCTAGCCAATGGCTCTTATC	acgttggatgTCCTTGTTTCTCTTGTGGGC	ATGGAAACCAGCGTGAT
